# Michigan State Board of Health

**Published:** 1882-08

**Authors:** 


### ARTICLE VIII.

### MICHIGAN STATE BOARD OF HEALTH.

The regular quarterly meeting of the State Board of Health was held July 11, at the office of the board in Lansing, the following members being present: Leroy Parker, of Flint, president; Rev. D. C. Jacokes, Pontiac; Drs. Henry F. Lyster, Detroit; J. H. Kellogg, Battle Creek; John Avery, Greenville, and Henry H. Baker, of Lansing, Secretary.

#### SMALL-POX AT FLINT.

Mr. Parker presented an account of an out-break of small-pox, from which, and a report made by Dr. Lyster, who had visited the case at Flint, it was determined that the first case was of a woman aged about fifty-five years, who traced her exposure to no other source than a peddler, supposed to have come from Canada, who had called at her house. Three weeks after the woman was taken sick, the daughter came down with the disease, and in three weeks more a boy, exposed by the daughter, was taken sick. This case was diagnosed as chicken-pox. The health officer diagnosed the case as small-pox, but met with violent opposition when he attempted to quarantine the family. Other cases appeared, called chicken-pox by some, and small-pox by others. Finally two physicians of Detroit went to Flint, and assured the citizens that the disease was small-pox, justifying the action of the health officer, who is now seconded in his efforts to restrict the disease. There are some fifteen cases of small-pox, and they are largely due to the willfulness of persons in trying to maintain that it was not small-pox, against the views of the health officer.

### BUILDING INSPECTION.

Dr. Kellogg reported, as a member of the State Board of Health and State Board of Charities, relative to the plans for a dormitory and school at the reform school, detailing the changes advised in the plans to make the building more perfect in a sanitary way.

Dr. Avery, as a member of the joint committee from the same boards to visit the reformatory at Ionia, reported that the warden assured the committee that the changes they had advised in the shops now under construction at that institution would be carried out.

### WORK OF THE OFFICE.

The secretary presented a detailed report of the work performed in the office during the last quarter, as follows: Much correspondence and hard work in starting the immigrant inspection service, which is now yielding valuable information, as well as aiding in preventing the spread of diseases. The inspectors at Port Huron and Detroit now make weekly reports to this board. Three cases of small-pox have been found on trains at Port

Huron, and many cases of measles have been found on trains between Port Huron and Detroit.

The various small documents printed by the board, for the prevention and restriction of diphtheria, scarlet fever, etc., and instructions as to the work of health officers in the restriction and prevention of various diseases, have been distributed to health officers throughout the State and a great many others, including masters and secretaries and lecturers of Michigan granges to the number of 840. These are sent in the hope that they will bring the subject before their organizations, and thus aid in disseminating information.

The returns of the names of 1,025 health officers in Michigan have been received, examined, and entered on the list, and a second demand sent for returns to such places as are yet dilatory. Correspondence has been larger than usual, on account of some new work in regard to immigrant inspection, etc.

A letter was read from the manufacturers of a nursing bottle which had been criticised by this board as poisonous, having a lead sinker attached to the rubber tube, stating that the objectionable feature would not hereafter be manufactured by them. They have substituted a glass sinker.

### CONTAGIOUS DISEASES.

A letter was received from J. Heitmann, health officer at Jamestown, Ottawa county, stating that scarlet fever was introduced into that township by immigrants from Holland, and eight deaths had occurred.

The secretary mentioned a report that scarlet fever was communicated to a cashier in a bank at Sault St. Marie, by money received from immigrants passing through there.

Dr. Lyster reported an outbreak of diphtheria in the upper peninsula, the disease having been brought in by immigrants.

A *résume* of the work of other State boards of health was read by the secretary.

### SANITARY CONVENTIONS.

An invitation for a sanitary convention at Reed City, signed by the editors, ministers, and doctors of that place, was accepted conditionally. The time for the convention was fixed about the last of November. The board also voted to accept an invitation and hold a sanitary convention at Pontiac in January.

NATIONAL BOARD OF HEALTH.

The danger to public health interests, caused by insufficient appropriations for the National Board of Health, was considered, and telegrams expressive of the apprehensions of this board in case the work of the National Board of Health was crippled, were sent to Michigan senators, and the president and secretary directed to forward a memorial to Congress on the subject.

CHEMICAL ANALYSES.

Dr. Lyster introduced a preamble and resolution, which was adopted, as follows:

"WHEREAS, It is essential to the health and well-being of the people of the commonwealth that all articles of food offered for sale should be free from adulteration;

"*Resolved*, That this board have such analyses and reports made, by experienced chemists, on such articles of food as may be submitted to them by the officers of this board, and that such sum of money as may be required, not exceeding $150 for the year 1882, be devoted to the necessary expense of such analyses."

Dr. Baker offered the following, which was adopted: "That the secretary be authorized to have analyses made of tissues, secretions, and excretions of the human body, to aid in determining the causes of certain diseases, at an expense not exceeding $100."

Dr. Lyster, special committee to report on the present knowledge respecting the cause and prevention of typhoid fever, read the introduction to a paper on this subject, which was accepted with thanks, and he was requested to complete the paper for publication in the report.

The examination of candidates in sanitary science was postponed until the October meeting of the board, which will occur October 10.

